# EEG and ECG-Based Multi-Sensor Fusion Computing for Real-Time Fatigue Driving Recognition Based on Feedback Mechanism

**DOI:** 10.3390/s23208386

**Published:** 2023-10-11

**Authors:** Ling Wang, Fangjie Song, Tie Hua Zhou, Jiayu Hao, Keun Ho Ryu

**Affiliations:** 1 Department of Computer Science and Technology, School of Computer Science, Northeast Electric Power University, Jilin 132013, China; smile2867ling@neepu.edu.cn (L.W.); 2202101005@neepu.edu.cn (F.S.); 2202100964@neepu.edu.cn (J.H.); 2 Data Science Laboratory, Faculty of Information Technology, Ton Duc Thang University, Ho Chi Minh City 700000, Vietnam; khryu@tdtu.edu.vn; 3 Biomedical Engineering Institute, Chiang Mai University, Chiang Mai 50200, Thailand; 4 Department of Computer Science, College of Electrical and Computer Engineering, Chungbuk National University, Cheongju 28644, Republic of Korea

**Keywords:** machine learning, EEG and ECG signal recognition, fatigue driving detection, feature extraction, real-time computing

## Abstract

A variety of technologies that could enhance driving safety are being actively explored, with the aim of reducing traffic accidents by accurately recognizing the driver’s state. In this field, three mainstream detection methods have been widely applied, namely visual monitoring, physiological indicator monitoring and vehicle behavior analysis. In order to achieve more accurate driver state recognition, we adopted a multi-sensor fusion approach. We monitored driver physiological signals, electroencephalogram (EEG) signals and electrocardiogram (ECG) signals to determine fatigue state, while an in-vehicle camera observed driver behavior and provided more information for driver state assessment. In addition, an outside camera was used to monitor vehicle position to determine whether there were any driving deviations due to distraction or fatigue. After a series of experimental validations, our research results showed that our multi-sensor approach exhibited good performance for driver state recognition. This study could provide a solid foundation and development direction for future in-depth driver state recognition research, which is expected to further improve road safety.

## 1. Introduction

Drivers can be distracted by cell phones while driving and may not notice situations occurring in front of them in enough time, thus increasing the risk of collisions. Some drivers suffer from impaired mental state due to driving for a long time or sleep deprivation, resulting in slow reactions to road situations, operational errors or momentary drowsiness, which greatly increases the probability of traffic accidents [1,2]. Therefore, the development of effective methods of identifying driver state could effectively reduce the probability of traffic accidents [3]. There are two main categories within fatigue driving detection research: video-based driving behavior detection and physiological signal detection.

Fatigue is a physiological state that leads to a series of changes in the body, which are manifested through physiological signals, such as eye movements, electroencephalogram (EEG) signals, heart rate variability and skin electrical activity [4,5,6,7,8]. By monitoring these physiological signals, we can indirectly gauge driver fatigue levels. Wang [9] established a brain function network-based detection method for fatigue driving using an EEG dataset. The correlations between EEG channels were calculated using partial directed coherence (PDC) and four graph-related features, including local efficiency, clustering coefficient, feature path length and node degree, were extracted. Then, SVMs were used as classifiers for fatigue detection. Qin [10] introduced a methodology for studying how driver fatigue affects the brain’s ability to process information, with a particular focus on EEG-based directed brain networks. By analyzing EEG signals using current source density (CSD) data, a directional brain network was constructed for the study of driver fatigue. Zhang [11] presented a system that utilized 14-channel EEG signals to effectively determine the fatigue influence degree in real time. This system used the sample entropy method to quantify complex EEG signals with SampEn features and a three-times support vector machine classifier (SCS model), which concluded that SampEn features could effectively distinguish between normal and fatigue EEG signals. Ren [12] presented a fatigue driving detection method using EEG signals and a hierarchical RBF network (RBF-TLLH) that enabled the global optimization of critical network parameters. Du [13] proposed a novel deep learning framework called Product Fuzzy Convolutional Network (PFCN) that could detect fatigue levels using an RBF neural network as a classifier. The PFCN method efficiently fused EEG signals and ECG signals and achieved high detection performance even under noisy conditions. Butkeviciute [14] used a machine learning algorithm combined with ECG signals for fatigue detection signal feature extraction, principal component analysis and classification. Halomoan [15] used a two-electrode ECG to measure fatigue state and employed heart rate variability resampling techniques and analysis to extract features, which were combined with an AdaBoost model for classification. Garcia-Perez [16] captured physiological signals from devices, extracted feature combinations and constructed machine learning-based evaluation models to classify different levels of sleepiness. Ebrahimian [17] introduced a driver sleepiness detection system, which leveraged a combination of CNN and LSTM. Additionally, it utilized HRV, respiratory rate and HRV power spectral density data to classify sleepiness levels. Dolezalek [18] determined heart rate, blink rate and brain activity by analyzing electromyography (EMG), electroencephalogram (EEG) and electrocardiogram (ECG) signals and then processed the different types of signals using filtering and correlation techniques. These rates were then compared to driver sleepiness levels to evaluate driver attention.

When a driver is fatigued, their facial expressions usually change. These changes may include more frequent blinking, longer eye closure and yawning. By analyzing facial expressions, it is possible to ascertain whether a driver is showing signs of fatigue [19,20]. Liu [21] used the RestNet10-SSD target detection algorithm to evaluate images of drivers’ faces, adopted Dlib tracking processing to enhance face detection and employed a fusion algorithm for face detection and tracking, which could effectively detect fatigue driving. Yin [22] used image recognition technology to extract the facial features of drivers from video-captured images and calculate eye aspect ratios as a means of determining driver state. Xiao [23] introduced a behavioral recognition approach employing Multi-Scale Facial Landmark Detector (MSFLD). This method consisted of deriving fatigue parameters from key facial features. To recognize fatigue driving, a hybrid approach combining adaptive and statistical thresholding was employed. Dong [24] used S3FD to detect faces within images and then extracted facial features using FAN to determine the driver yawn rate, head posture and eye state. In the final step, the random forest technique was employed for driving condition analysis. In parallel, a CNN was utilized to classify the various types of distracted driving. Zhou, F. [25] used PERCLOS (i.e., percentage of eyelid closure time) as a basic fatigue indicator, and thus, a predictive model was developed to predict fatigue using these key physiological characteristics. Wang F. [26] proposed a driving fatigue detection method based on a multiple nonlinear feature fusion strategy. Six widely used nonlinear features of EEG signals were used for feature extraction. A Multi Kernel Learning (MKL) support vector machine fuses and classifies these extracted features. Additionally, the best recognition accuracy of 81.33% was achieved.

Current mainstream research methods have achieved promising results, proving the feasibility of recognizing driver fatigue state based on physiological signals and images. Among the EEG-based studies, researchers have tended to use large numbers of channels for state recognition in order to improve accuracy, while multi-signal fusion research has been relatively scarce and needs further investigation. We propose a simplified EEG channel for fatigue state recognition that uses a small number of channels, which could be more conducive to use with portable EEG acquisition devices. This channel could also be used to fuse multi-sensor signal sources and construct real-time feedback models.

This paper structure is as follows: Section 2 describes the materials and methods; Section 2.2 presents the EEG-based fatigue driving detection model; Section 2.3 describes the ECG-based fatigue driving detection model; Section 2.4 introduces the image-based distracted driving detection model; Section 2.5 discusses the proposed real-time feedback model; Section 3 presents our evaluation of the experimental results and any shortcomings; Section 4 describes the direction of future work; Section 5 summarizes the study and results.

## 2. Materials and Methods

During fatigue driving detection, the use of a single detection method often leads to incorrect judgments, so we combined EEG signals and ECG signals to detect fatigue driving. In addition, considering real driving environments, we chose to use a convenient EEG acquisition device. We only used two-channel EEG data for the EEG-based fatigue driving detection and recognition and achieved a high accuracy rate. In order to identify abnormal driving behaviors, driver cell phone use and lane deviations were detected using image recognition technology.

This study carried out more in-depth research on abnormal driving and we were able to detect fatigue driving using EEG and ECG signals. We used dual-channel EEG signals for detection, which are more suitable for portable mobile applications. We also detected driver cell phone use and lane deviations using image recognition technology. Finally, we evaluated real-time driver state responses using a real-time feedback system, which could significantly bolster the advancement of mobile application development.

**Figure 1:**
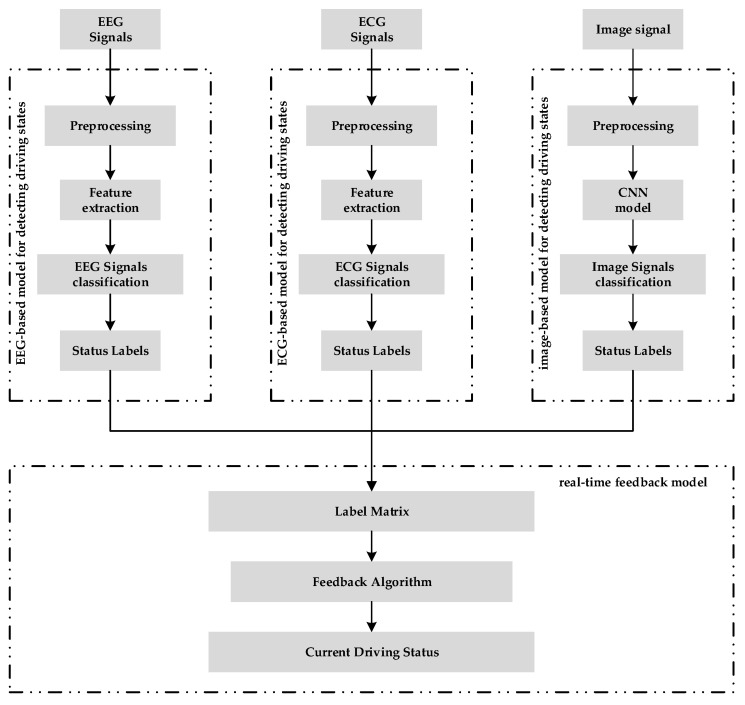
Overall framework.

Figure 1 shows our overall framework. The entire process comprised the following key models: the EEG model, which detected fatigue driving by extracting eight EEG signal features; the ECG model, which recognized fatigue driving by calculating HRV features; the image-based model, which detected driver cell phone use and lane deviations using in-vehicle and external cameras. Finally, we combined the results from the three models and input them into a feedback model to provide real-time feedback on driver status.

### 2.1. Datasets

The first EEG dataset comprised raw EEG data from 12 healthy participants and included two states (please refer to [27]). The second EEG dataset comprised data from eight male and eight female healthy participants (please refer to [28]). These two datasets were applied to EEG-based detection of fatigued driving. The DROZY ECG dataset was also used in this study; this dataset includes polysomnographic signals from 14 healthy participants (please refer to [29]). This dataset is applied to detect fatigued driving based on ECG. The distracted driver detection and classification dataset contains a large amount of image data, which demonstrate various driver behaviors, such as cell phone use, etc. (please refer to [30]). The TuSimple dataset includes a large number of high-resolution images and annotated information regarding real driving scenarios, which can be used for lane line detection and lane departure detection. This dataset can also be used to train and test vehicle deviation detection algorithms (please refer to [31]).

### 2.2. EEG-Based Fatigue Driving Detection Model (EE-FRE Model)

The EEG-based fatigue driving detection model is our proposed model for detecting fatigue driving. Raw EEG signals are susceptible to environmental and physiological factors during the acquisition process, so noise reduction techniques are required. In this study, considering the subsequent application of this model in practical situations, we did not use a complex noise reduction process. We used standard pre-processing only. We just filtered the signals through a 0.15 Hz low-pass filter to remove low-frequency noise. Additionally, we used a 40 Hz high-pass filter to eliminate high-frequency noise, along with a 50 Hz notch filter to mitigate power interference. 

#### 2.2.1. Feature Extraction

EEG signals can be converted into frequency domain representations using the Fourier transform. Spectral characterization means that EEG signals can be analyzed in the frequency domain by decomposing the levels using the Fourier transform. Table 1 shows comprehensive details regarding the frequency ranges and the standard EEG frequency bands.

EEG signals in the time domain can be converted into frequency representations using a fast Fourier transform to obtain information from different frequency domains. By calculating the area under the curve, we can determine the energy of EEG signals in different frequency bands (e.g., δ-wave, θ-wave, α-wave, β-wave, etc.).

EEG signals are complex and irregular, so the classification and identification of EEG signals are realized by calculating their entropy values signals in different states.

Fuzzy entropy serves as an indicator for evaluating signal complexity. It combines the concepts of fuzzy theory and entropy and is used to describe nonlinear characteristics and dynamic changes in EEG signals.

(1) $$\mathrm{Fuzzy}\;\mathrm{Entropy}=-\sum (p\left(i\right)\times \log\left(p\left(i\right)\right)),$$

where $p\left(i\right)$ is the fuzzy probability distribution of the signal, which is calculated by combining the signal’s membership functions with fuzzy set theory.

Sample entropy is a measure of the degree of signal irregularity and complexity. In EEG signal analysis, sample entropy can be used to assess the nonlinear characteristics and complexity of signals. It is also often used to study dynamic changes and instability in brain activity.

(2) $$\mathrm{Sample}\;\mathrm{Entropy}=-\log(\frac{A}{B}),$$

where *A* represents the number of subsequences with a signal length of m that exhibit a distance from other subsequences of the same length that is less than the specified threshold r and *B* stands for the number of subsequences with a signal length of m − 1 that exhibit a distance from other subsequences of the same length that is less than the prescribed threshold r. In this context, m signifies the chosen subsequence length and r signifies the designated threshold value.

Spectral entropy is used to reveal changes in the spectrum of EEG signals, the characteristics of frequency band activity and the interrelationships between different frequency bands by calculating the statistical properties of energy distributions and spectral patterns.

(3) $$\mathrm{Spectral}\;\mathrm{Entropy}=-\sum (p\left(f\right)\times \log\left(p\left(f\right)\right)),$$

where p(f) is the power spectral density of the signal at frequency f, which can be calculated by conducting a spectrum analysis of the signal.

Approximate entropy calculates entropy values by evaluating the degree of similarity between repetitive patterns with lengths of m and m + 1 in EEG signals. It is used to compare signal properties in different states.

(4) $$\mathrm{Approximate}\;\mathrm{Entropy}=-\log(\frac{C(m,R+1)}{C(m-1,r+1)}),$$

where C(m, r + 1) signifies the number of subsequences with a signal length of m that exhibit a distance from other subsequences of the same length that is less than the specified threshold r and C(m − 1, r + 1) represents the number of subsequences with a signal length of m − 1 that exhibit a distance from other subsequences of the same length that is less than the prescribed threshold r. Here, m denotes the chosen subsequence length and r denotes the designated threshold value.

**Figure 2:**
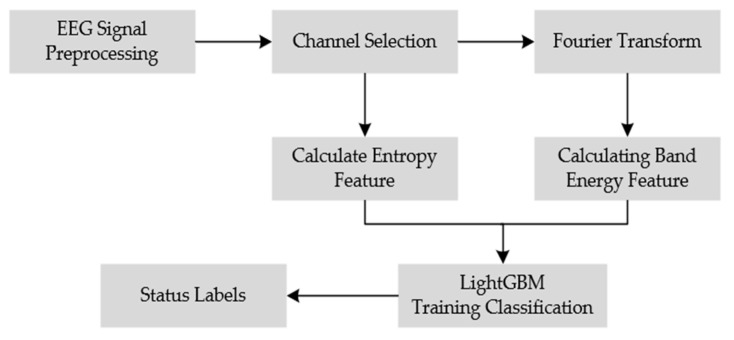
EEG-based fatigue driving detection model.

The employed EEG dataset contained 32 channels of EEG data for fatigue driving detection using portable devices, so two channels (P3 and P4) of data were selected for our experiments. These two electrodes are located in the posterior region of the brain, which is the area associated with the cognitive functions of the brain die cut, and at the same time this area facilitates the acquisition of data by the sensors. Our model employs the LightGBM machine learning framework [32], an integrated learning method based on gradient boosting trees. Its main advantages are high performance and low memory usage. The complete EEG-based model for fatigue driving detection is depicted in Figure 2.

#### 2.2.2. EEG-Based Model for Detecting Driver State

Regarding the extraction of genuine EEG-based driver state recognition features, the specified parameters are presented in Table 2.

The detailed EEG fatigue recognition engine is shown in Algorithm 1.
**Algorithm 1:** EEG Fatigue Recognition Engine**Input**: EES **Output**: classification accuracy *Acc* 1: **Begin** 2: import EES to python 3: Read the EES and select the signal of two of the channels4: X ← EES; H ← 0.15 Hz; L ← 40 Hz; D ← 50 Hz; 5: **for** each of EES **do**6:  PEES = bandpass filtering(X, H, L);7:  PEES = depressionfiltering(PEES, D);8: **end for**9: **for** each of PPS **do**10:  FF = calculate frequency (fourier transform(PEES));11:  EF = calculate entropy (PPS); 12: **end for** 13: train_label, train_data = Select 80% of data(FF + EF); 14: test_label, test_data = Select 20% of data(FF + EF);14: model = LightGBMtrain (train_label, train_data);15: classification accuracy as *Acc* = LightGBMdict(test_label, test_data, model);

### 2.3. ECG-Based Fatigue Driving Detection Model (EC-FRE Model)

The ECG-based fatigue driving detection model is our proposed model for detecting fatigue driving. To remove noise from the ECG data, signals from 0.1 to 30 Hz were bandpass filtered and detrended to remove the baseline offset.

#### 2.3.1. Feature Extraction

The R-wave detection algorithm was used to find the peak points of R-waves in the ECG signals and thus extract the features.

The following calculation formulae offer a more detailed description of our interpretation of ECG characteristics.

RMSSD is an indicator of heart rate variability and is used to measure the degree of variability between adjacent RR interval differences. We determined the total by summing the squares of the differences between neighboring RR intervals. Subsequently, this value was divided by (N − 1) before we performed the root mean square operation.

(5) $$\mathrm{RMSSD}=sqrt(\frac{sum({\left(RR\left[i+1\right]-RR[i]\right)}^{2})}{N-1}),$$

where $RR\left[i\right]$ represents the ith RR interval and N denotes the RR interval.

AVRR is the average value of RR intervals, which was utilized to compute the mean heart rate. To obtain this average, we summed all RR interval values and divided the result by the total number of RR intervals.

(6) $$\mathrm{ARVV}=\frac{sum\left(RR\right)}{N},$$

where RR denotes all RR intervals.

SDRR, which stands for the standard deviation of RR intervals, serves as a metric for quantifying the extent of RR interval variability. To calculate SDRR, we initially computed the sum of the squared differences between each RR interval and ARVV. Subsequently, this sum was divided by (N − 1) as part of the standard deviation computation.

(7) $$\mathrm{SDRR}=sqrt(\frac{sum({\left(RR\left[i\right]-AVRR\right)}^{2})}{N-1}),$$

SKEW refers to the skewness of RR interval distributions, which is used to measure the symmetry of RR interval distributions. The cubic sum of the differences between each RR interval and AVRR was calculated and divided by (N ∗ SDRR^3^).

(8) $$\mathrm{SKEW}=sum\left(\frac{RR[i]-{AVRR}^{3}}{N*{SDRR}^{3}}\right)$$

KURT is the kurtosis of RR interval distributions and is used to measure the sharpness of RR interval distributions. We summed the squared differences between each RR interval and the mean RR interval and then divided the result by (N ∗ SDRR4) before subtracting three.

(9) $$\mathrm{KURT}=sum\left(\frac{RR\left[i\right]-{AVRR}^{4}}{N*{SDRR}^{4}}\right)-3$$

**Figure 3:**
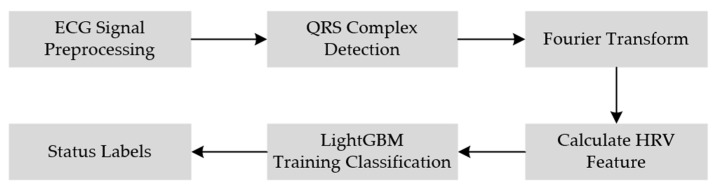
ECG-based fatigue driving detection model.

Using the method for feature extraction from ECG signals detailed above, the complete ECG-based model for fatigue driving detection is depicted in Figure 3.

#### 2.3.2. ECG-Based Model for Detecting Driver State

Regarding the extraction of genuine ECG-based driver state recognition features, the specified parameters are presented in Table 3.

The detailed EEG fatigue recognition engine is shown in Algorithm 2.
**Algorithm 2:** ECG Fatigue Recognition Engine**Input**: ECS **Output**: classification accuracy *Acc* 1: **Begin** 2: import ECS to python3: Y ← ECS; Hi ← 0.1 Hz; Lo ← 30 Hz; T ← 0.3; 4: PECS = bandpass filtering (Y, Hi, Lo); 5: PECS = Trend processing (PECS); 6: QRS_Filter = sin (1.5 ∗ π, 3.5 ∗π 15); 7: **for** each of PECS **do**
8:  SIM = correlate (PECS, QRS_Filter) 9:  **if** SIM > T 10:   RI = index (PECS); 11:   HF = calculate HRV feature (RI); 12:  **end if** 13: **end for** 14: train_label, train_data, test_label, test_data = Select 80% of data(HF); 15: test_label, test_data = Select 20% of data(HF); 16: model = LightGBM train (train_label, train_data); 17: classification accuracy as *Acc* = LightGBM dict (test_label, test_data, model);

### 2.4. Image-Based Abnormal Driving Detection 

Our proposed image-based abnormal driving detection model used deep learning to process images via a convolutional neural network (CNN), which is a deep-learning architecture that is exceptionally well suited to image processing tasks. In contrast to traditional image processing methods, CNNs can automatically learn image features without the need to manually design and select features; therefore, they are more efficient and accurate for processing complex image data.

The model contained a series of convolutional, pooling and fully connected layers. The combination of these layers allowed the model to gradually extract features from images and achieve image classification in the final fully connected layer. The first layer was a convolutional layer that contained 32 3 × 3 filters and a ReLU activation function. Subsequently, the first pooling layer reduced the size of the feature map via a maximum pooling operation, which preserved crucial features. This was followed by a second convolutional layer, which contained 64 3 × 3 filters and a ReLU activation function. The second pooling layer also applied a maximum pooling operation. Lastly, the third convolutional layer contained 128 3 × 3 filters and a ReLU activation function. In order to classify the features, we added a flattening layer to the architecture of the model, which spread the output of the final convolutional layer into a one-dimensional vector in preparation for the fully connected layers. After this stage, there were two fully connected layers. The initial fully connected layer comprised 128 neurons and the second fully connected layer comprised 64 neurons. ReLU activation functions were utilized by both of these layers. The final fully connected output layer contained 192 neurons. For multicategory classification, we employed the softmax activation function.

When training the model, we utilized the Adam optimizer to optimize the parameters of the model. The model’s loss was calculated using the sparse classification cross-entropy loss function. Finally, we chose accuracy as the evaluation criteria. Our model achieved an accuracy rate of 96%.

### 2.5. Real-Time Feedback Model

A real-time feedback model was then implemented based on the above models. To implement the real-time feedback model, we first set up a dynamic matrix to store the labels generated by the other models. This dynamic matrix played a key role in the model feedback process and was constructed gradually using inputs from each sample. This real-time update feature allowed us to easily record and analyze the model outputs, which in turn allowed for further real-time feedback and adjustments. The rows of this matrix represented the labels of each sub-event, while the columns represented the labels of different sub-events within the same time series.

**Figure 4:**
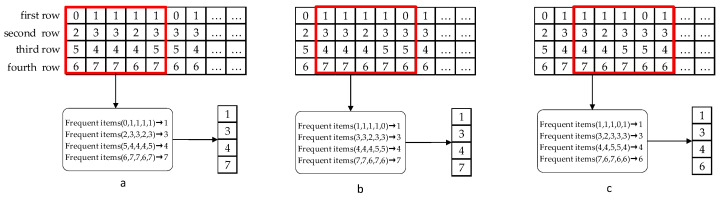
Sliding window calculation process.

As shown in Figure 4a, in the first row, label 1 indicates the detection of driver fatigue through EEG signals, while 0 represents the driver being in a non-fatigued state. In the second row, label 3 signifies the detection of driver fatigue through ECG signals, whereas 2 indicates the driver being in a non-fatigued state. In the third row, label 5 denotes the detection of driver phone usage, while label 4 signifies the driver not using a phone. In the fourth row, label 6 indicates that the vehicle is driving normally within the lane, while label 7 represents a lane departure situation.

Then, we used a sliding window (with a window size of 5 s) to perform real-time analysis of the continuously incoming data stream. The sliding window moves one column per second to continuously analyze the data, as shown in Figure 4b,c. Within the window, we need to calculate the most frequently occurring item in each column of data. For instance, in Figure 4a, we calculate the data within the sliding window as follows: the first row of data (0, 1, 1, 1, 1) has the most frequent item as 1, and the second row (2, 3, 3, 2, 3) has the most frequent item as 3, continuously, resulting in a pattern (1, 3, 4, 7). As the window slides, we continuously calculate new modes and update the results. The graphical representation is shown in Figure 4.

**Figure 5:**
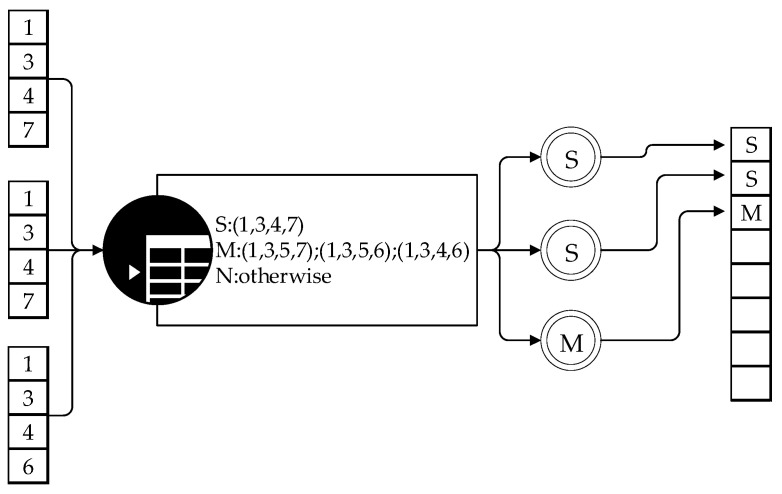
Feedback calculation process.

To ensure that the algorithm can handle various scenarios, we need to pre-establish a dictionary that includes all possible label patterns and their corresponding outcomes. In this dictionary processing, as an example, (1, 3, 4, 7) represent the severity fatigue level and (1, 3, 5, 7), (1, 3, 5, 6) and (1, 3, 4, 6) represent the mild fatigue level, and the others are normal driving situations, as shown in Figure 5. In this study, there are four types: EEG, ECG, image recognition (hand-phone detection and lane departure detection). There is a potential possibility that if driver decides to “make or receive a phone” call, it may lead to “lane departure”, and that means the driver may not be engaging in fatigued driving. But if the driver does NOT “make or receive a phone” call, and “lane departure” occurs, and the same time, one of the EEG or ECG signals seems abnormal, it may present a risk. For this situation, we select a severity level which demonstrates abnormal external expression. Otherwise, if only EEG or ECG signals detect the fatigued driving status, the fatigue is deemed to be mild.

Whenever calculating a mode pattern, we begin by checking if there is a matching key in the dictionary. When a match is found, it will return the corresponding result. This mapping of the label pattern to the dictionary is a critical step in the algorithm, as it helps us map the label modes obtained by analyzing the data to predefined results in the dictionary. The calculation flowchart is shown in Figure 5.

Finally, to calculate the mapped result, we use the following formula:

(10) $$R=\left\{\begin{array}{c} “\mathrm{Severe}\;\mathrm{fatigue}”,if\;s=1\;and\;∆t>T;\\ “\mathrm{Mild}\;\mathrm{fatigue}”,if\;m=1\;and\;∆t>T;\\ “normal”,otherwise \end{array}\right.$$

In this context, “*s*” and “*m*” are keys in the dictionary, representing severe fatigue driving and mild fatigue driving, respectively. “∆*t*” represents the status and end time slides when the mapping results appeared, and “*T*” is the presetting threshold. This formula takes the mapped label pattern and performs the necessary calculations to generate the ultimate feedback result.

In summary, the real-time feedback model could efficiently process and analyze real-time data streams by applying multiple models, setting up dynamic matrices and using sliding windows and a dynamic programming algorithm. This integrated application allowed the model to better adapt to complex and changing data situations and provide accurate real-time feedback.

## 3. Experiment and Results

The computers employed in this experiment featured Xeon 2620 processors with a main clock speed of 2.1 GHz, accompanied by NVIDIA GTX 1080 Ti GPUs. The software environment used was Anaconda. In our experiments, we considered five datasets: the Figshare dataset [27], the Mendeley dataset [28], the DROZY dataset [29], the Distraction Detection dataset [30], and the TuSimple dataset [31], which contains 53,705 volumes’ data in total. We divided datasets into 80% for training and 20% for testing. For further details, please see Section 2.1.

### 3.1. EE-FRE Model Tests

EEG-based modeling is a significant method for fatigue detection as it can aid in determining whether a driver is experiencing fatigue, thus effectively improving road traffic safety. In this study, we used a two-channel EEG (electroencephalogram) dataset, which reported driver EEG signals when driving.

We extracted two types of features from EEG signals, namely frequency features and entropy features. Frequency features reflect the energy distributions of EEG signals in different frequency bands, while entropy features describe the complexity and randomness of EEG signals. By independently evaluating the use of frequency features and entropy features, we found that both types of features performed well in fatigue driving detection, achieving accuracy rates of 79% and 77%, respectively. To further enhance the accuracy of fatigue driving recognition, we combined frequency and entropy features and constructed a comprehensive model. The results indicated that our proposed comprehensive model attained a satisfactory accuracy of 90.17% on the two-channel EEG dataset. This indicated that the amalgamation of these features could significantly enhance the fatigue driving detection model performance.

**Figure 6:**
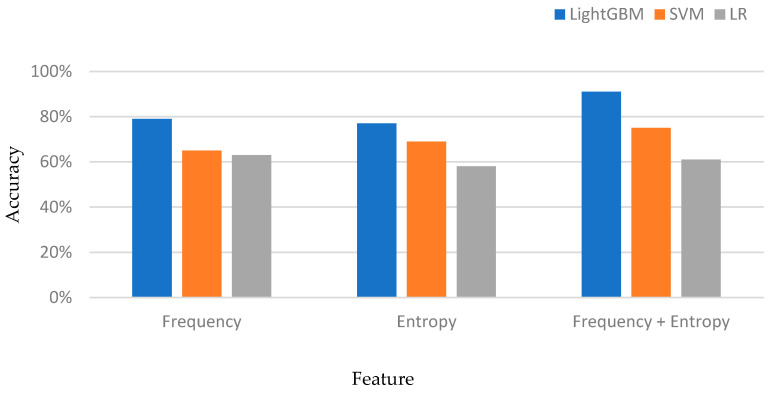
Feature extraction performance comparison.

We also carried out comparative experiments using traditional SVM classifiers [33] and LR logistic regression models [34] in order to validate the effectiveness of our proposed model. The results are presented in Figure 6.

**Figure 7:**
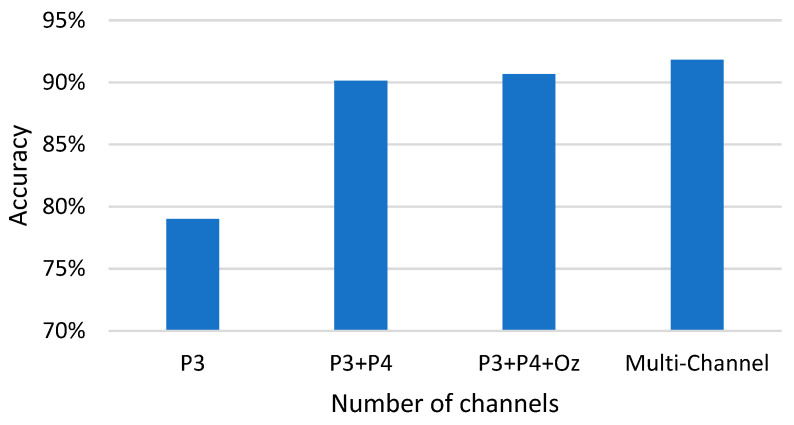
Comparison of accuracy using different numbers of channels.

In addition to combining different types of features into our fatigue driving detection model to improve accuracy, we also considered how to reduce the number of channels while maintaining high accuracy in the case of using convenient EEG acquisition devices to improve model portability and utility. A series of experiments was conducted, including single-channel, dual-channel, and three-channel EEG data, as well as multi-channel EEG data, to evaluate the performance of the model. The results showed that using dual-channel EEG data produced the best balance between accuracy and portability. This is important for fatigue driving monitoring in real-life scenarios, as EEG acquisition devices usually need to be worn on the head, so a smaller number of channels could simplify the structure of the device, reduce wearer discomfort and improve driver acceptance. The experimental results are shown in Figure 7.

**Figure 8:**
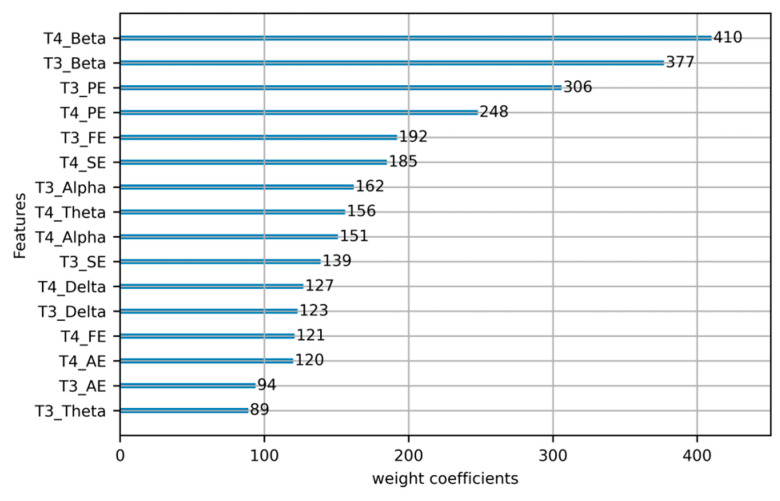
EEG Feature weight coefficients.

To gain a deeper understanding of the importance of EEG features in fatigue driving detection, we also calculated scores for different features. The feature weight coefficients plot is shown in Figure 8.

**Figure 9:**
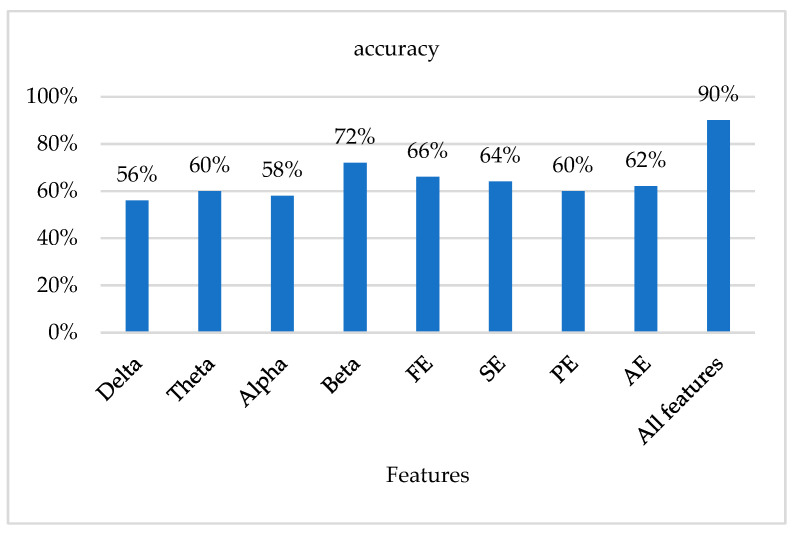
Single and multi-features fusion performance comparison.

We conducted repeated experiments, testing each feature individually, and the experimental comparisons are shown in Figure 9.

**Figure 10:**
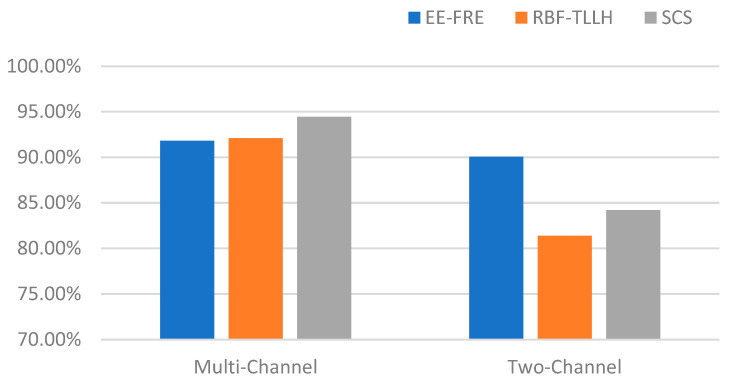
Comparison of accuracy using different models under different conditions.

To evaluate model performance, we compared our method to the RBF-TLLH method [12] and the SCS model [11] using the previously described dataset. The experiments showed that all methods were effective in feature extraction and model training when dealing with complex and diverse data. This implied that all methods would be worthy of consideration when resources allow. Nevertheless, it is worth highlighting that our proposed model exhibited superior performance when only utilizing two-channel data. This indicated that our model could produce a better performance under the given constraints (i.e., fewer data and more limited information) and although the performances of the compared methods were similar on multichannel data, our proposed model performed better on two-channel data when resources were limited. This result could provide strong support for the selection of our model in specific application scenarios. The experimental results are shown in Figure 10.

### 3.2. EC-FRE Model Tests

ECG-based fatigue driving detection is now widely recognized as an effective method for assessing driver state using biological information from ECG signals. In this study, we focused on the extraction of HRV features and after experimental validation, our model achieved an excellent performance using the LightGBM classifier, with an accuracy rate of 92%. This indicated that the extracted HRV features had strong discriminative ability in fatigue driving detection and that the LightGBM classifier could effectively utilize these features to accurately determine driver fatigue levels.

**Figure 11:**
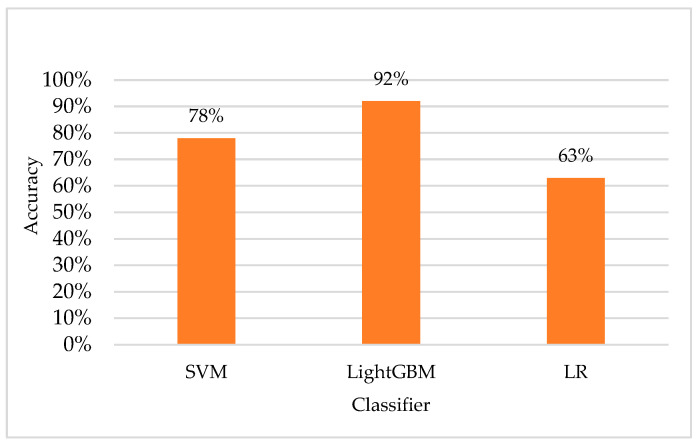
Feature performance using different classifiers.

In addition, we also conducted comparative experiments using a traditional SVM classifier and an LR logistic regression model. SVMs are common classifiers that usually perform well in many applications. However, it is interesting to note that in this study, the SVM performed poorly in fatigue driving detection, with an accuracy rate of only 78%. Similarly, the LR model only achieved an accuracy of 63%. Figure 11 clearly shows the difference in performance between the models and highlights the excellent performance of our proposed fatigue driving detection model.

**Figure 12:**
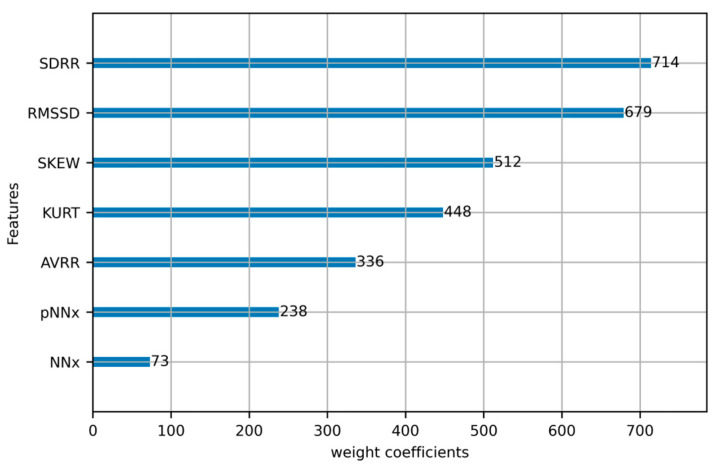
ECG Feature weight coefficients.

To further understand the importance of individual features in fatigue driving detection, we also calculated scores for different features. These scores helped us to understand which HRV features played key roles in accurate fatigue driving detection and helped us to optimize model performance and feature selection. The experimental results are shown in Figure 12.

**Figure 13:**
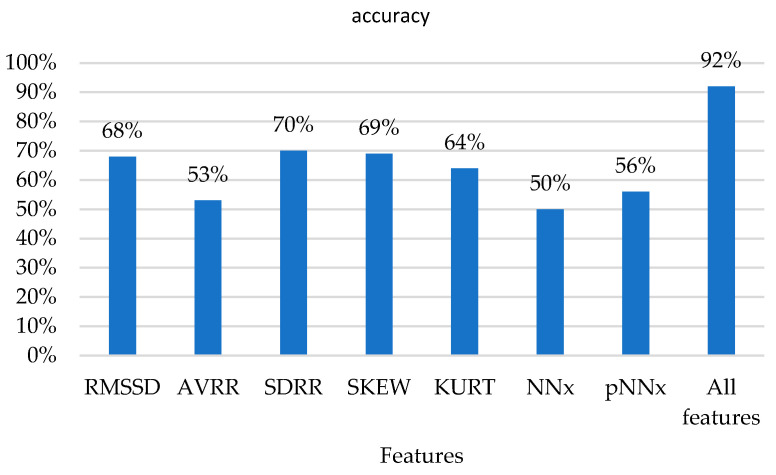
Experimental comparative chart.

At the same time, we conducted multiple experiments, testing the model’s performance using each feature separately, and the experimental comparisons are shown in Figure 13.

**Figure 14:**
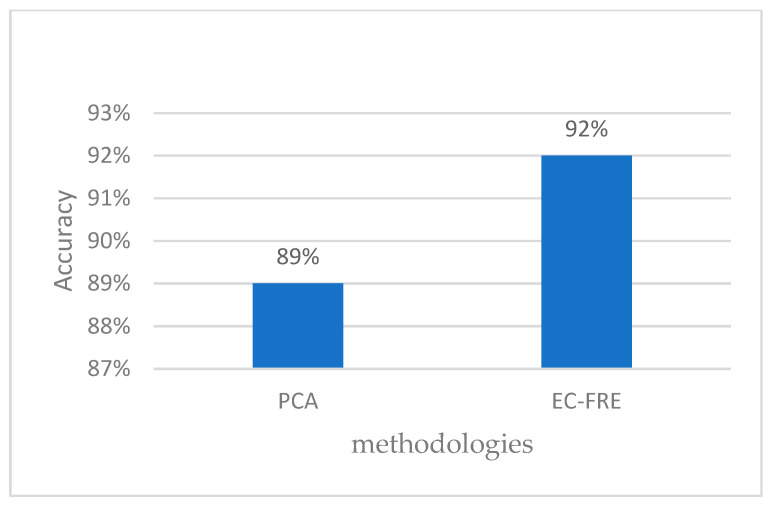
Comparison of model accuracy.

In this section, we evaluate the classification effectiveness of our proposed electrocardiogram (ECG) signal-based model. For comparison, we chose to use the state-of-the-art model presented by Wang. as a benchmark, which has achieved significant results in previous studies. After performing an experimental evaluation, we found that our method achieved some improvements compared with the model presented by Wang. The experimental results are shown in Figure 14.

## 4. Discussion

In this study, we proposed a multi-sensors fusion computing for real-time fatigue driving recognition which contains three types of signals (EEG, ECG, Videos) and four kinds of data types (EEG P3 and P4 channels, HRV features, phone calls behavior, and lane departure detection). After deep analysis of the correlations among these data types’ features, a real-time feedback model was proposed to judge the real driving fatigue status and give the fatigue severity levels. EEG P3 and P4 channels selection is limited by portable devices consideration, which is more comfortable and helpful for drivers’ daily usage. ECG signals detection is more suitable for driver monitoring fatigue-induced heart diseases or emotion-induced related diseases that occur when driving, and this form of detection will be more widely used in the future. EEG and ECG are biosensors that track the driving status based on the use of portable devices, and image-based detection is the external expression evaluation of the fatigue severity level; the detailed logic calculation is shown in Section 2.5.

Although our model was trained and tested using a variety of driving simulator datasets that offer realistic results for dynamic scenarios, we are well aware that there are differences between simulations and real driving environments. In order to better approximate real driving scenarios, we have decided to conduct further measures to compensate for this discrepancy in future studies. The plan is to actively collect data from real driving environments in specific areas, such as roads, city blocks and motorways. This could allow us to capture more complex and diverse driving behaviors and situations and obtain more accurate reflections of real-world driver states. Combining data from real driving environments and simulations could help to enhance a model’s ability to generalize and make it more adaptable to various road conditions and driving styles. This type of data fusion approach could improve the performance and credibility of our model in real driving scenarios.

When analyzing driver state using images, the datasets employed in this study contained well-lit images; however, we fully recognize that in real road environments, driving can be affected by weather and lighting conditions. In order to better address these challenges and ensure that our model performs well under all conditions, we have opted to investigate the utilization of infrared cameras in future research. By using infrared cameras, we could bypass the interference of conventional cameras in both bright and low-light environments, resulting in more stable and reliable image data and allowing us to ensure that driver state and behaviors could still be accurately identified under complex lighting conditions.

During the construction of the real-time feedback model, we set up four different events for real-time feedback. After obtaining labels for the different signals, we input different events into the feedback model; however, in subsequent studies, we aim to evaluate the connections between signal labels and events and create more events, thereby improving the model.

It is also important to highlight that in order to study driver state, invasive methods are used to acquire EEG and ECG signals, which can hinder the driver’s free movement during experiments and can result in subconscious reactions that inevitably cause driver discomfort. In this study, we used EEG signals to recognize the driver state, but we were able to employ a smaller number of channels while maintaining accuracy. We hope that this technique can be widely applied to portable devices, which could make it more likely for drivers to accept the use of these devices.

Our goal is to build a more robust and reliable driver state analysis model to facilitate the development of smart driving technology and ultimately contribute to safer, more efficient and smarter road traffic systems. We will continue to make further efforts to explore new data collection methods and techniques to improve our research results and the effectiveness of our model.

## 5. Conclusions

In this study, we overcame the limitations of single-signal methods by fusing multiple sources to achieve the research objective of recognizing driver state. The EEG and ECG modeling experiments showed that high accuracy rates could be obtained when distinguishing driver states using our selected extracted features. Therefore, our EEG and ECG models achieved better driver state recognition. In addition, in order for our model to be applicable to portable devices, we only used two channels in EEG signal acquisition and processing to judge driver state, which achieved a high accuracy rate.

Our experimental results showed that the CNN- and image-based driver state recognition model we designed could effectively recognize driver cell phone use and lane deviations. In the final part of our research, we designed a real-time feedback model. This model was designed and realized based on our other experimental models. The collected signals were processed using the other models and then the results were input into the real-time feedback model, which could evaluate driver state information in real time. In the feedback model, we explored different conditions, such as driver fatigue, driver cell phone use, etc.

Our research could contribute to the development of multi-sensor fusion technologies for driver state recognition, as well as laying foundations and providing further directions for subsequent research.

## Figures and Tables

**Table 1:** EEG signal frequencies.

Frequency Band	Frequency Range (Hz)
δ	0–4
θ	4–8
α	8–12
β	12–30

**Table 2:** EEG algorithm notations.

Parameter	Definition
EES	Raw EEG signal
H	High-pass filter
L	Low-pass filter
D	Notch filter
PEES	Preprocessed signal
FF	Frequency feature
EF	Entropy feature

**Table 3:** ECG algorithm notations.

Parameter	Definition
ECS	Raw ECG signal
Hi	High-pass filter
Lo	Low-pass filter
PECS	Preprocessed signal
T	Similarity threshold
QRS_Filter	Filter for calculating QRS waves
SIM	Similarity calculation
RI	R-wave sequence
HF	HRV feature

## Data Availability

A publicly available dataset was analyzed in this study. The data can be found here: https://data.mendeley.com/datasets/dpgvc22rth/1 (accessed on 19 February 2019).
